# Design of the multi-material structure using an MMC-SIMP sequential topology optimization method

**DOI:** 10.1371/journal.pone.0321100

**Published:** 2025-05-09

**Authors:** Zhao Li, Hongyu Xu, Shuai Zhang, Jintao Cui, Xiaofeng Liu

**Affiliations:** 1 School of Mechatronics Engineering, Henan University of Science and Technology, Luoyang, China; 2 School of Vehicle and Traffic Engineering, Henan University of Science and Technology, Luoyang, China; TU Dublin Blanchardstown Campus: Technological University Dublin—Blanchardstown Campus, IRELAND

## Abstract

The present paper introduces a sequential topology optimization method for multi-material structure design. In the proposed method, the moving morphable component (MMC) method and the solid isotropic material with penalization (SIMP) method are performed in sequence to achieve multi-material structure optimization. First, the structural topology is obtained using the MMC method, and then the material layout of the determined structural topology is optimized using the SIMP method. The connection between MMC method and SIMP method is established through components mapping elements, where changes in the material layout within these elements do not affect the external structural topology. The proposed method effectively combines the advantages of two optimization methods, enabling the achievement of an explicit external structural topology and greater freedom in internal multi-material layouts, while remaining easy to implement. The key MATLAB codes for the proposed method are provided in the paper. Finally, the feasibility of the proposed method is verified through some numerical examples.

## 1. Introduction

Compared with single-material structures, multi-material structures can make more rational use of materials with different properties to jointly bear structural loads and meet various design requirements. Additionally, multi-material structures can further enhance the lightweight level of the structure and provide a broader range of lightweight design ideas. Particularly with the rapid development of additive manufacturing technology [1–3], it has become increasingly convenient to manufacture multi-material structures at a relatively low cost. As a result, multi-material structural design has attracted extensive attention.

In order to make more effective use of multi-material structures, it has become increasingly urgent to develop efficient multi-material structural design methods. Topology optimization, which seeks the optimal materials layout within a predetermined design domain to meet structural performance requirements, has been widely and profoundly developed [4–9]. In recent years, the realization of multi-material structural design using topology optimization methods has gradually become a research hotspot [10–12]. Compared to the single-material topology optimization method, which only determines the presence or absence of materials in the design domain, multi-material topology optimization can be regarded as a problem of determining the material state of each point in the design domain. In other words, the optimal layout of multiple materials in the structure can be achieved by determining whether there are materials and what materials exist at each point in the design domain. The core content of multi-material topology optimization lies in how to achieve parameterization of materials. The key issue is to construct a so-called material interpolation mechanism to represent material property indicator functions (such as density function in SIMP method, level set function in level set method, and component description function in MMC method). Bendsøe and Sigmund [13] introduced the most commonly used hybrid interpolation scheme, which uses power functions to interpolate between holes and materials. Wang et al. [14] proposed a multi-material level set interpolation scheme, where each material is represented by a combination of different level set functions instead of a specific level set function. Additionally, the parameterized level set method [15–17] also has potential for application in topology optimization of multi-material structures, which core idea is to convert partial differential equations into ordinary differential equations, thereby eliminating a series of numerical difficulties in solving partial differential equations. Zhang et al. [18] proposed a multi-material description method. When the optimization problem involves multiple types of solid materials, under the guidance of the basic idea of the MMC method, multiple sets of components are introduced, and all components in the same set have the same material properties (including elastic modulus, Poisson’s ratio, etc.).

Commonly speaking, the design variables in multi-material topology optimization methods will significantly increase after introducing multi-material parameters, which has a significant impact on computational cost. To reduce computational cost, Wei and Wang [19] proposed a segmented constant level set method, which uses an index function containing *m* predefined constants to represent the distribution of *m* materials. Although this method can reduce the number of design variables, an additional highly non-convex equality constraint must be introduced to ensure that the index function converges to the predefined segmented constants. Zuo and Saitou [20] established an ordered SIMP interpolation scheme, which interpolates the elastic modulus and cost properties of multiple materials with respect to normalized density variables by introducing a power function containing scaling and translation coefficients, without introducing new variables. Based on the ordered SIMP interpolation scheme, Xu et al. [21] proposed a method for solving stress constrained multi-material topology optimization (SMMTO) problems. In this method, ordered SIMP interpolation functions are used to achieve relaxation and scaling stress interpolation, so the SMMTO problem only requires a unique set of density variables. Park and Sutradhar [22] used a multi decomposition algorithm to handle three-dimensional multi material topology optimization problems, mainly by using an alternating “active phase” method to divide the problem into several “material-void phase” topology optimization problems, and *m*-materials optimization problem can be divided in m*(*m*-1)/2 sub problems. Based on the material-field series expansion (MFSE) model [23,24], Bao et al. [25] proposed an effective topological representation and dimensionality reduction method. In this method, a specified number of material phases is described within a single material field with a piecewise Heaviside projection function, and the number of design variables is independent of both the number of material phases and the FE mesh. Although the above method can reduce the number of design variables to a certain extent, it still requires the introduction of some topological description functions defined throughout the entire design domain to describe the distribution of multiple materials. Moreover, when incorporating multiple materials into the topology design of structural systems, the optimization model and its corresponding solution process will become more complex due to the interaction of multi-material phases in the ultra-high dimensional design space.

To solve the above-mentioned problems, it is necessary to develop a simple and highly applicable multi-material topology optimization method. Among various topology optimization methods, implicit topology optimization methods are relatively mature, and explicit topology optimization method can significantly reduce design variables in some cases and can more accurately extract boundary information. However, the comprehensive use of explicit and implicit topology optimization methods can fully leverage their respective advantages. To seek the optimal distribution of materials, Deng [26] proposed a two-step topology optimization method. First, a rough material configuration is obtained based on the traditional density method. Then, the inverse optimization problem is solved to fit the geometric components to the solution obtained in the previous step, resulting in a good optimization response. Li et al. [27] developed an integrated optimization strategy that includes two different topology optimization methods: the MMC method for predicting the initial topology structure and the SIMP method for refining the subsequent topology structure, optimizing the microchannel layout in multi-channel heat sinks. Guo et al. [28] proposed a hybrid explicit implicit topology optimization method for the design of shell-infill composite structure, by using MMC method to optimize the shell and SIMP method to optimize the internal filling structure.

In this paper, a sequential topology optimization method based on the MMC method and the SIMP method is proposed to achieve multi-material structure design in a simple and feasible way. Compared with a single optimization method, the proposed method can fully leverage the advantages of different optimization methods. The MMC method has an explicit boundary expression, while the SIMP method offers higher degrees of freedom in multi-material selection. Compared with existing hybrid optimization methods, the proposed method can improve computational efficiency and is easier to converge and implement. The remaining sections of the paper are organized as follows: The principles of sequential topology optimization method are introduced in Section 2. The problem formulation and numerical implementation are described in Section 3. The key MATLAB codes of the proposed method are explained in Section 4. In Section 5 and Section 6, the effectiveness of the proposed method is verified through several numerical examples. Finally, some concluding remarks are given.

## 2. MMC-SIMP sequential topology optimization method

The principle of the MMC-SIMP sequential topology optimization method is to determine the optimal structural topology (force transmission path) using the MMC method, and then optimize the material layout within the known structural topology to obtain a multi-material structure.

### 2.1. MMC-SIMP sequential topology optimization flow

**Fig 1:**
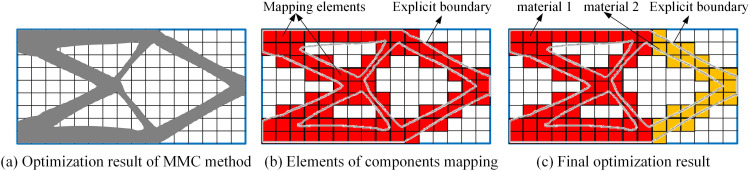
MMC-SIMP sequential topology optimization method.

The optimization flow of the MMC-SIMP sequential topology optimization method can be summarized as follows:

(1) Obtain the topology optimization result based on the MMC method, and determine all component topology description functions $\phi^{S}(x)$ , as shown in Fig 1a.
(2) Find the elements $e=[e_{1},\cdots ,e_{i},\cdots ,e_{m}]$ under $\phi^{S}(x)$ mapping, where *m* is the total number of mapping elements, including solid elements and boundary elements, as shown in Fig 1b.
(3) Construct a material selection model, determine the design variables of SIMP method, and select materials for element $e_{i}$.
(4) Perform finite element analysis and sensitivity analysis.
(5) Update the design variables of SIMP method.
(6) Check for convergence.
(7) Obtain multi-material structure, as shown in Fig 1c.

### 2.2. MMC method for structural topology

In the MMC method, a set of moving morphable components are adopted as the basic building blocks of topology optimization. These components are allowed to deform, move, overlap and merge in the design domain freely, and structural topology can be obtained by changing the positions, inclined angles, lengths, widths and the layout of these components. Moreover, these components contain explicit geometric parameter information, which can be directly evolved to optimize structural topology. The structural topology description of the MMC method can be achieved in the following way:


$$\left\{ \begin{array}{cc} @ll\phi^{S}(x)>0, & if\;x\in \Omega^{S}\\ \phi^{S}(x)=0, & if\;x\in \partial \Omega^{S}\\ \phi^{S}(x)<0, & if\;x\in D/(\Omega^{S}\cup \partial \Omega^{S}) \end{array}\right.$$ (1)

where *D* is a prescribed design domain, $\Omega^{S}$ is the area occupied by solid materials in the design domain, $\partial \Omega^{S}$ represents the boundary and $D\backslash (\Omega^{S}\cup \partial \Omega^{S})$ is the void area. $\phi^{S}(x)$ is the topological description function of all components, which can be expressed as:


$$\phi^{S}(x)=\max({\phi_{1}}^{e},\cdot \cdot \cdot ,{\phi_{i}}^{e},\cdot \cdot \cdot ,{\phi_{n}}^{e})$$ (2)

where ${\phi_{i}}^{e}$ is the area occupied by the *i*-th component, which can be described as:


$$\left\{ \begin{array}{cc} @ll\phi_{i}^{e}(x)>0, & if\;x\in \Omega^{i}\\ \phi_{i}^{e}(x)=0, & f\;x\in \partial \Omega^{i}\\ \phi_{i}^{e}(x)<0, & f\;x\in D/(\Omega^{i}\cup \partial \Omega^{i}) \end{array}\right.$$ (3)

### 2.3. Component mapping elements identification

Based on the surrogate material model, component mapping elements can be identified. The surrogate material model describes the density of elements based on the topological function values of nodes and divides them into void density elements, intermediate density elements, and solid density elements. Taking the four-node bilinear element as an example, the element density of the void element is 0, the element density of the intermediate element are [1/4, 2/4, 3/4], and the element density of the solid element is 1. Therefore, the topology optimization method based on the surrogate material model only needs to identify elements with a density greater than 0, which are the component mapping elements.

The surrogate material model can be represented as:


$$\rho_{e}^{M}=\frac{\sum_{i=1}^{4}}{{\left(H(\phi_{i}^{e})\right)}^{q}4}$$ (4)

where *H = H(x)* is the Heaviside function and $\phi_{i}^{e}$, *i* = 1,…,4 are the values of the topology description function of the whole structure at four nodes of element *e*. *q* is the penalty factor (usually *q* = 2). In order to ensure the stability of numerical implementation, *H(x)* is usually regularized in the following form:


$$H_{\varepsilon }(x)=\left\{ \begin{array}{cc} @lc1, & x>\varepsilon \\ \frac{3(1-\alpha )}{4}\left(\frac{x}{\tau }-\frac{x^{3}}{3\tau^{3}}\right)+\frac{1+\alpha }{2} & -\varepsilon \leq x\leq \varepsilon \\ \alpha , & x<-\varepsilon \end{array}\right.$$ (5)

where ε is a regularization parameter that controls the width of the smooth transition of the Heaviside function, and α is a small positive number used to simulate the stiffness of void structure in surrogate material models, in order to avoid singular phenomena in the overall stiffness matrix of the structure. Therefore, for component mapping elements, the element density should be greater than $\alpha^{q}$.

### 2.4. SIMP method for material selection

The surrogate material model is only used to identify whether elements contain materials, and subsequently material selection is required for the elements containing materials. Taking two-phase materials as an example, the following model can be utilized for element material selection.


$$E=\rho_{e}^{p}E_{1}+(1-\rho_{e}^{p})E_{2}$$ (6)

where *ρ*_*e*_ is the artificial density of the material, *p* is a penalization power (generally *p* ≥ 3), *E*_*1*_ and *E*_*2*_ are the Young’s moduli of material 1 and material 2, respectively. The material interpolation model does not contain voids, *E*_*1*_ represents the base material, *E*_*2*_ represents the reinforcement material, and the layout of the reinforcement material is determined by the design variable *ρ*_*e*_.

## 3. Problem formulation and numerical implementation

The MMC-SIMP sequential topology optimization method is based on the principle of sequence to complete the design of multi-material structures. The proposed method involves two sets of solving processes, including: MMC optimization and SIMP optimization, with the key being to achieve data linkage between the two optimization methods. The MMC method is used to obtain initial data (solid material regions), and the specific implementation method can be referred to in [29]. On this basis, material selection is achieved through the SIMP method. In this paper, the formulation and numerical implementation of SIMP problems are primarily analyzed.

### 3.1. Problem formulation

Taking the minimum flexibility of the structure as the objective function and considering the total volume of the structure and the volume ratio of each material relative to the base material as constraints, the problem formulation can be described as follows:


$$\begin{array}{c} \begin{array}{cc} & Find\;D={\left(\rho_{1},\rho_{2}...,\rho_{NE}\right)}^{T}\\ & Minimize\;C=U^{T}KU=\sum_{e=1}^{NE}E_{e}\left(\rho_{e}\right)u_{e}^{T}k_{0}u_{e}\\ & subject\;to\left\{ \;\;\;\begin{array}{c} F=KU\\ \sum_{i=1}^{m}V_{i}=V(D),\;m=1,2,3...\\ C_{i}/V_{1}=f_{1} \end{array}\right. \end{array} \end{array}$$ (7)

where *D* is the material selection density variable, and its value is the number of component mapping elements *NE*. *C = U*^*T*^*KU* is the structural flexibility, *u*_*e*_ and *k*_*0*_ are the element displacement and the element stiffness matrix without Young’s modulus respectively, *E*_*e*_*(ρ*_*e*_*)* is the element density function. *F = KU* is the governing equation of finite element analysis. *V(D)* is the total volume of the structure. *V*_*i*_ is the *i*-th material volume with $\sum_{i=1}^{m}V_{i}=V(D),\;m=1,2,3...$. *V*_*1*_ is the base material, and *f*_*i*_ is the volume ratio of the *i*-th material to the base material. The design domain of SIMP is the topological structure mapping domain *V(D)* of MMC optimization results, and $V(Dle\bar{V}$, where $\bar{V}$ is the volume constraint of the overall structure. Taking two-phase materials as an example, the volume constraint should also meet $V_{1}+V_{2}=V(D)$ and $V_{2}=V_{1}*f$. In the implementation algorithm, the volume constraint of two-phase materials can be realized by constraining *V*_*1*_.

### 3.2. Finite element analysis

The key in finite element analysis is to calculate the overall stiffness matrix. Based on the topological optimization method proposed in this paper, taking two-phase materials as an example, the element stiffness matrix can be expressed as:


$$k^{e}=\rho_{e}^{M}[\rho_{e}^{p}E_{1}+(1-\rho_{e}^{p})E_{2}]k_{0}$$ (8)

The overall stiffness matrix is


$$K=\sum_{e=1}^{NE}k^{e}$$ (9)

### 3.3. Sensitivity analysis

Sensitivity of objective function can be expressed as:


$$\frac{\partial_{ke}}{\partial \rho_{e}}=\rho_{e}^{M}[p\rho_{e}^{p-1}(E_{1}-E_{2})]k_{0}$$ (10)

According to the constraint conditions, the volume of material 1 (the ratio of the volume occupied by the material 1 to the design domain volume) is


$$V_{1}=\frac{1}{DW*DH}\sum_{e=1}^{NE}\rho_{e}^{M}\rho_{e}*EW*EH$$ (11)

where *DW* and *DH* are the length and width of the design domain respectively, *EW* and *EH* are the length and width of the element respectively.

The total volume of the structure can be expressed as:


$$V(D)=V_{1}+V_{2}=(1+f)V_{1}=\frac{1+f}{DW*DH}\sum_{e=1}^{NE}\rho_{e}^{M}\rho_{e}*EW*EH$$ (12)

where *f=V*_*2*_*/V*_*1*_。

The sensitivity of constraint condition can be expressed as:


$$\frac{\partial V(D)}{\partial \rho_{e}}=\frac{1+f}{DW*DH}*\rho_{e}^{M}*EW*EH$$ (13)

In order to avoid the checkerboard phenomenon and reduce the mesh dependence, the sensitivity filtering technology proposed by Sigmund [30,31] is adopted. The weighted average of the sensitivity of each element within the filtering radius is used to replace the sensitivity value of the central element. The modified sensitivity of the objective function to the design variables is


$$\frac{\partial \hat{c}}{\partial x_{e}}=\frac{\sum_{i=1}^{N}{\hat{H}}_{i}x_{i}\frac{\partial c}{\partial x_{i}}}{x_{e}\sum_{i=1}^{N}{\hat{H}}_{i}}$$ (14)

where ${\hat{H}}_{i}$ is the convolution operator (weight factor), which can be expressed as:


$${\hat{H}}_{i}=r_{\min}-dist(e,i),\;\left\{i\in NE,|dist\text{(}e,i\text{)}\leq {\text{r}}_{\min}\right\}$$ (15)

where *dist(e, i)* is the distance between the centers of element *e* and *i*, *r*_*min*_ is the filtering radius, and *NE* is the total number of elements.

## 4. Optimization algorithm

In this paper, the optimization algorithm of the SIMP method is mainly explained, including component mapping elements identification, finite element analysis, and sensitivity analysis. Component mapping elements identification is used to determine the position and number of mapping elements and assign initial density values. The relevant code is as follows

**Table:** 

Ei = find(denk>alpha^2); % Find the component mapping elements nn=length(Ei); % Determine the number of component mapping elements Svariable(1:nn,1)=volfrac; % Initial variables assignment Sxy00 = Svariable(:);

Global analysis is still adopted in the finite element analysis, where the Young’s modulus of the mapping elements is replaced according to the material selection model. The overall stiffness matrix is then assembled, and the global displacement is calculated. Finally, the new flexibility can be obtained. The relevant code is as follows.

**Table:** 

SE = Sxy00.^penal*E1+(1-Sxy00.^penal)*E2; % *Material selection model* *Ndenk = denk(:).^E1; %* *Initial Young’s modulus assignment E1 for all elements* *%* *Introducing material selection model to mapping elements* *for ia = 1:nn* Ndenk(Ei(ia))=denk(Ei(ia))*SE(ia); *end* *%* *Finite element analysis* NsK = NKE(:)*Ndenk(:)‘; *NK = sparse(iK(:),jK(:),NsK(:));* *NK = (NK + NK’)/2;* NU = zeros(2*(nely+1)*(nely+1),1); *NU(freedofs,:) = NK(freedofs,freedofs)\F(freedofs,:);* Comp01 = F’*NU;

Sensitivity analysis calculates only the density sensitivity information for material selection of the mapping elements. However, for the convenience of sensitivity filtering, the sensitivity information of other elements is defined as 0. Additionally, since two-phase materials are considered in this study, the optimization criterion method can be employed to update the variables. The relevant code is as follows.

**Table:** 

dc=zeros(nn,1); % Define sensitivity matrix % Calculate the sensitivity of all variables for ib = 1:nn UU = NU(edofMat(Ei(ib),:)); dc(ib)=penal* Sxy00(ib).^(penal-1)*(E1-E2)* UU’*NKE* UU; end col(1:nely,1:nelx)=0; col = col(:); for ic = 1:nn col(Ei(ic))=Sxy00(ic); end col = reshape(col,nely,nelx); % Sensitivity filtering rmin = 2; dc01=zeros(nely*nelx,1); for, i.e., = 1:nn dc01(Ei(i.e.,))=dc(i.e.,); end dc01 = reshape(dc01,nely,nelx); dc01 = Sfilter(nelx,nely,rmin,col,dc01); dc02 = dc01(:); dc03 = zeros(nn,1); for ig = 1:nn dc03(ig)=dc02(Ei(ig)); end % Variables update Sxy00 = OC(nn,Sxy00,f,dc03);

## 5. Numerical examples

### 5.1. Problem examples

**Fig 2:**
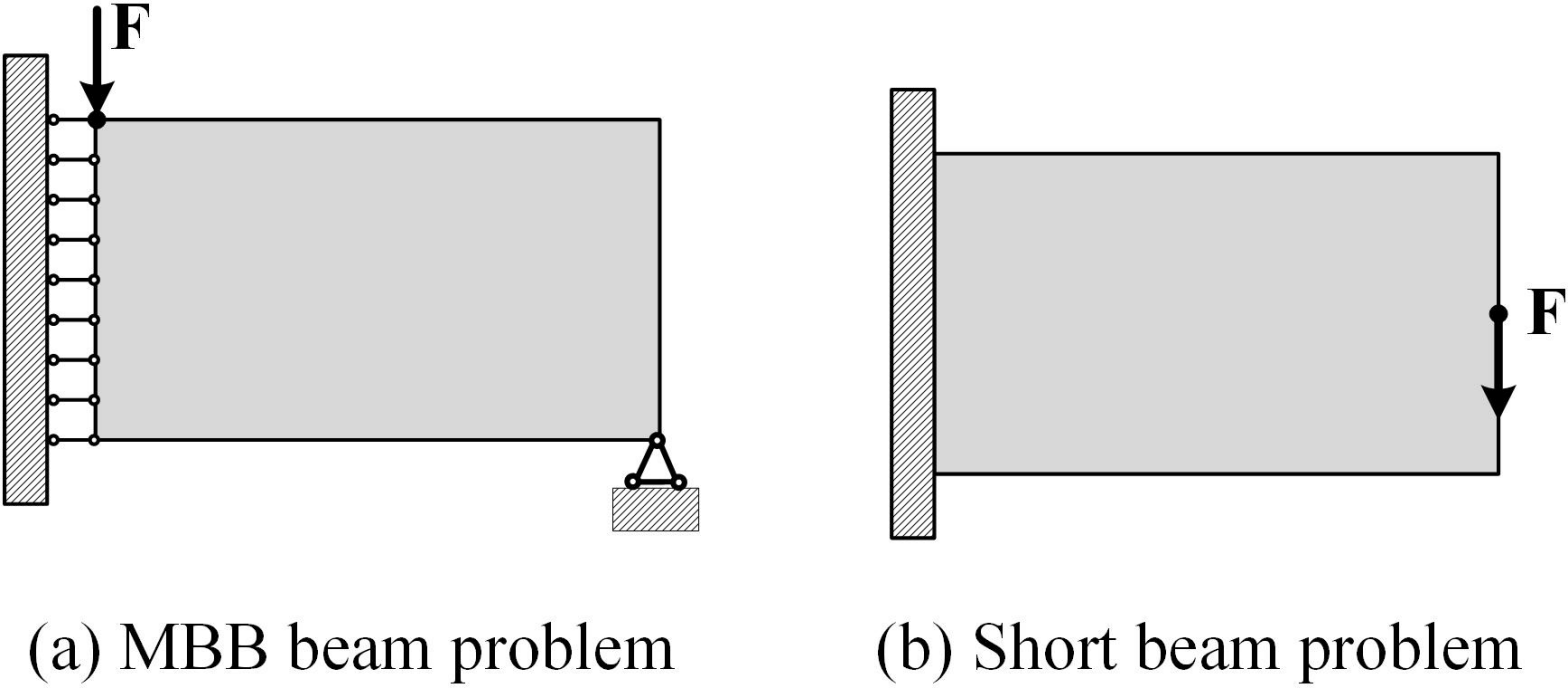
Problem examples.

In this section, the short beam problem and the MBB beam problem are selected as examples, as shown in Fig 2. The minimum flexibility of the structure is taken as the objective function in both numerical examples, and the total volume of the structure and the volume ratio of each material relative to the base material are taken as constraints. To simplify the analysis, it is assumed that all relevant quantities in the studied problems are dimensionless, and the thickness of all design domains is set to a unit value. Young’s modulus of two materials are set to *E*_*1*_ = 1 and *E*_*2*_ = 100, Poisson’s ratio is chosen as *v*_*s*_ = 0.3, total volume constraint is chosen as *V(D)*=0.4, volume ratios of two materials are set to *f* = *V*_*2*_/*V*_*1*_=[0.2, 0.5, 0.8, 1, 1.5, 2]. The load is set to *F* = 1. The penalization powers are chosen as *p* = 3 and *q* = 2, respectively.

### 5.2. Component description of MMC method

**Fig 3:**
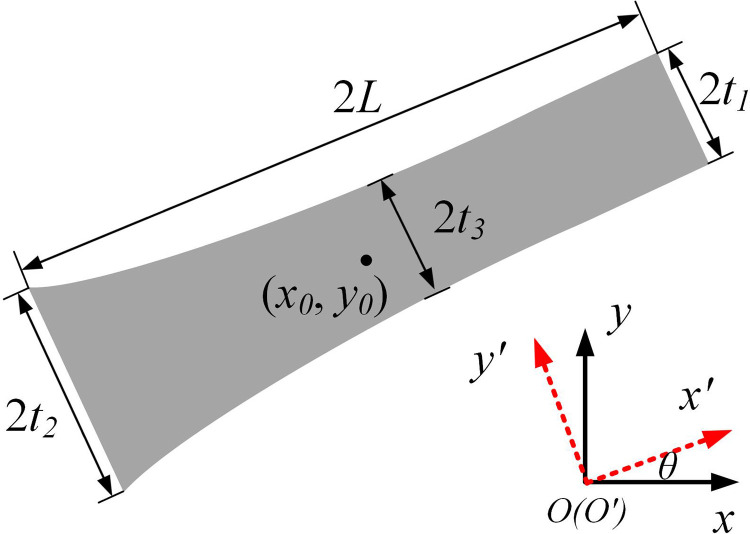
Component description.


In the MMC method, a quadratically varying thickness component is adopted, as shown in Fig 3, and by using hyperellipsoid equations, the component description function can be expressed as

$$\phi (x,y)=1-{\left(\frac{x^{\prime }}{L}\right)}^{p}-{\left(\frac{y^{\prime }}{f(x^{\prime })}\right)}^{p}$$ (16)

with


$$\left\{\begin{array}{c} x^{\prime }\\ y^{\prime } \end{array}\right\}=\left[\begin{array}{cc} \cos\theta & \sin\theta \\ -\sin\theta & \cos\theta \end{array}\right]\left\{\begin{array}{cc} & x-x_{0}\\ & y-y_{0} \end{array}\right\}$$ (17)

where ϕ(x,y) represents the topological function values of the coordinates corresponding to the whole design domain, and the parameter *p* is a relatively large positive even number (generally *p*=6). *L* is the half length of the component,($x^{\prime }$, $y^{\prime }$) and (*x*, *y*) represent the coordinates of any point in the local coordinate system and the global coordinate system respectively, *θ* is the rotation angle of the component (from the global coordinate system to the local coordinate system), $f(x^{\prime })$ is a function describing the shape of the component, which can be expressed as:


$$f(x^{\prime })=\frac{t_{1}+t_{2}-2t_{3}}{2L^{2}}{\left(x^{\prime }\right)}^{2}+\frac{t_{2}-t_{1}}{2L}x^{\prime }+t_{3}$$ (18)

## 6. Example analysis

Both the short beam problem and the MBB beam problem use the quadratically varying thickness component to achieve MMC topology optimization. The design domain size for the short beam problem is set to 2 × 1, and the mesh resolution is set to 80 × 40. The design domain size for the MBB beam problem is set to 3 × 1, and the mesh resolution is set to 90 × 30. In addition, a single hole thin-walled structural component (with scaling factor of *a* = *b* = 0.5) from reference [32] is selected to optimize the short beam problem, resulting in a thin-walled short beam structure. The thin-walled short beam problem remains as shown in Fig 2b, with a design domain size of 2 × 1 and a mesh resolution of 120 × 60. In the final optimized structure, the black area within the boundary represents the layout of material *E*_*1*_, and the green area represents the layout of material *E*_*2*_.

### 6.1. MBB beam problem

(1) **Optimization results**

**Fig 4:**
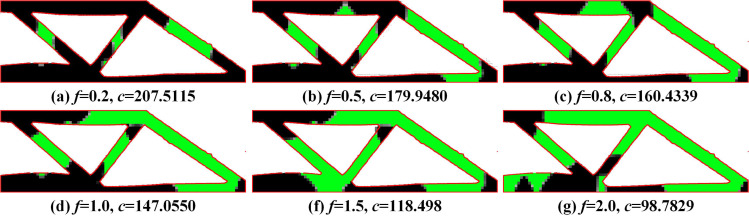
Optimization results of MBB beam problem under different *f.*

The optimization results of MBB beams under different *f* are shown in Fig 4. It can be seen that the proposed method can achieve a secondary material layout for the optimization results of the MMC method, further improving structural stiffness. Due to the implementation of secondary material layout based on the density method, it can not only leverage the high design freedom of the density method to generate more flexible material layouts but also exhibit better adaptability to complex structures. The proposed method has clear external structural topology boundaries and explicit expression, as well as a distinct internal multi-material layout, with fewer numerical issues such as checkerboard patterns and grayscale elements.

**Fig 5:**
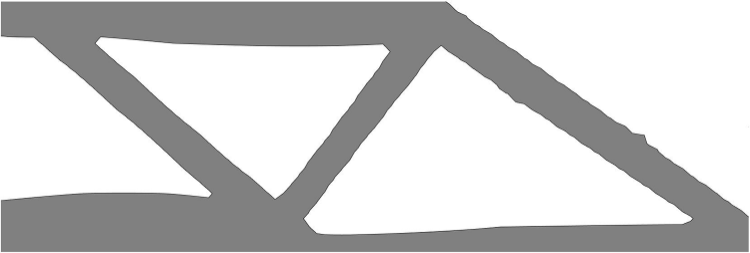
Single-material optimization results of MBB beam (*f*=0, *c*=230.147).

Compared with the single material topology optimization structure (as shown in Fig 5), the multi-material optimization structure significantly improves the structural stiffness.

**Fig 6:**
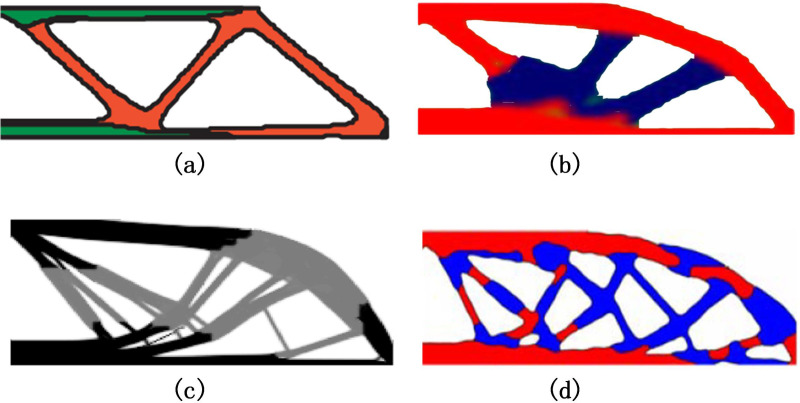
Optimization results of MBB multi-material structure under different methods. (a) A level-set method for multi-material topology optimization [33]. (b) An alternating active-phase algorithm for multi-material topology optimization [34]. (c) A new mapping based interpolation function for multi-material topology optimization [35]. (d) A material-field series expansion (MFSE) model for multi-material topology optimization [36].

Fig 6 shows partial optimization results obtained from other literature on the MBB beam multi-material problem. It can be seen that the proposed method also yields the clear multi-material structure and has a relatively stable topology with flexible material selection.

(2) **The influence of different Young’s modulus ratio**

**Fig 7:**
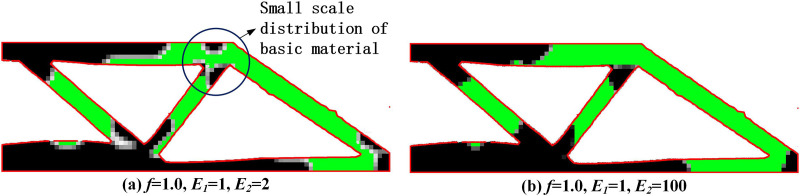
Optimization results under different Young's modulus ratio.

In order to calculate structural response using finite element method, the main topology optimization methods often assume that the void is a weak material, while the Young’s modulus of solid materials is much higher than that of weak material. In this way, when updating variables, the influence of weak material can be ignored to obtain feasible force transmission paths. Based on this, we found that when the difference in Young’s modulus between the two-phase materials is small (as shown in Fig 7a, where *E*_*1*_ = 1 and *E*_*2*_ = 2), the optimization results show that the basic phase material *E*_*1*_ has larger small-scale distributions. The reason is that considering the small difference in properties between the two-phase materials, the sensitivity difference during optimization is small. Although the optimization results are theoretically feasible, they have poor machinability. In practical multi-material structure design, the reinforcing phase material is often significantly better than the base phase material. When the Young’s modulus of the reinforcing phase material is much higher than that of the base phase, the small-scale distribution problem is significantly improved, as shown in Fig 7b.

### 6.2. Short beam problem

**Fig 8:**
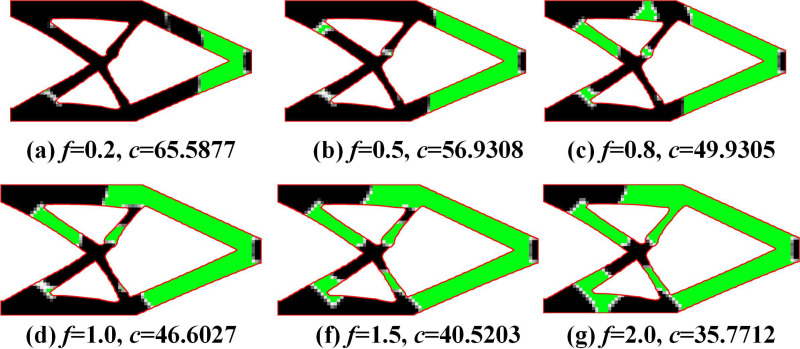
Optimization results of short beam problem under different *f.*

**Fig 9:**
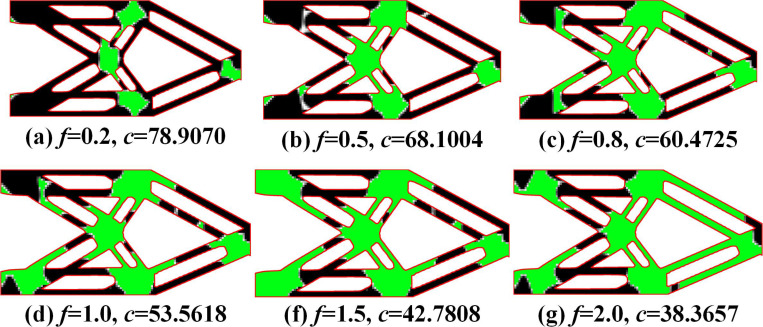
Optimization results of thin-walled short beam problem under different *f*.

**Fig 10:**
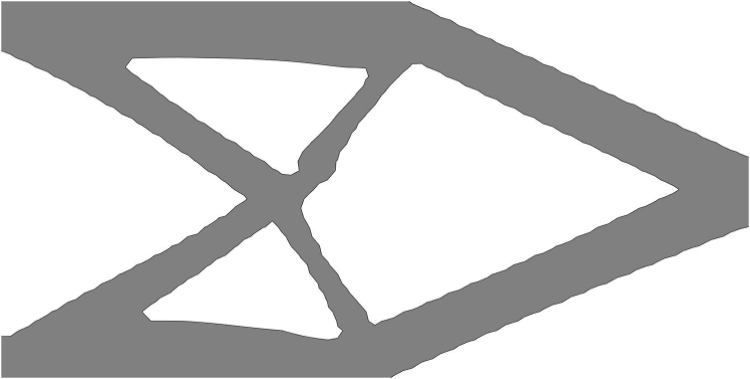
Single-material optimization results of short beam (*f*=0, *c*=75.4630).

**Fig 11:**
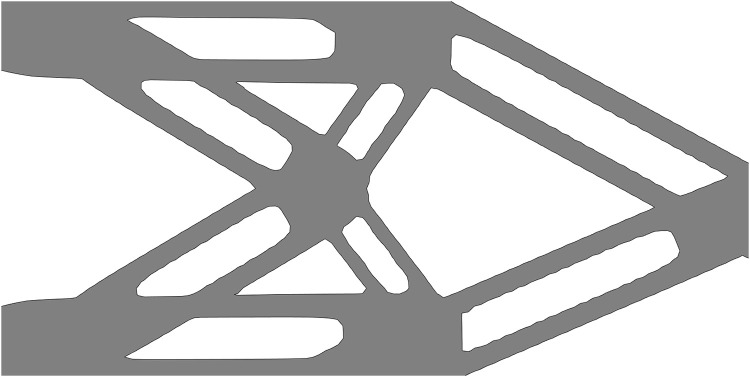
Single-material optimization results of thin-wall short beam (*f*=0, *c*=85.575).

To further verify the feasibility of the proposed method, the optimization results of the short beam problem and the thin-walled short beam problem are shown in Figs 8 and 9, and the optimization result of the single-material short beam problem and the thin-walled short beam problem are shown in Figs 10 and 11. It can be seen that the proposed method can achieve good multi-material layout in both structural problems, and the structural stiffness is superior to that of single-material structures (*E* = 1). The case of thin-walled short beams further demonstrates that the proposed method remains effective even as the complexity of the components increases.

Generally speaking, the performance of the reinforcement material in one aspect is better than that of the base material. In this paper, the material with a higher Young’s modulus is selected as the reinforcement material to reduce the flexibility of the structure, while the single material refers to the material with a lower Young’s modulus. Additionally, it should be noted that the advantages of multi-material structures can be fully utilized only after a reasonable layout of multiple materials; therefore, how to achieve a reasonable multi-material layout is the goal pursued by various topology optimization methods.

### 6.3. Optimization effect analysis

(1) **Flexibility reduction rate**

Introduce the flexibility reduction rate to analyze the optimization effect in different examples. The flexibility reduction rate can be expressed as:


$$\eta =\frac{C(f_{0})-C(f_{i})}{C(f_{0})}\times 100\%$$ (19)

where *C(f*_*0*_*)* is the structural flexibility at *f*_*0 *_= 0, and *C(f*_*i*_*)* is the structural flexibility at *f*_*i*_ = [0.2, 0.5, 0.8, 1.0, 1.5, 2.0].

**Fig 12:**
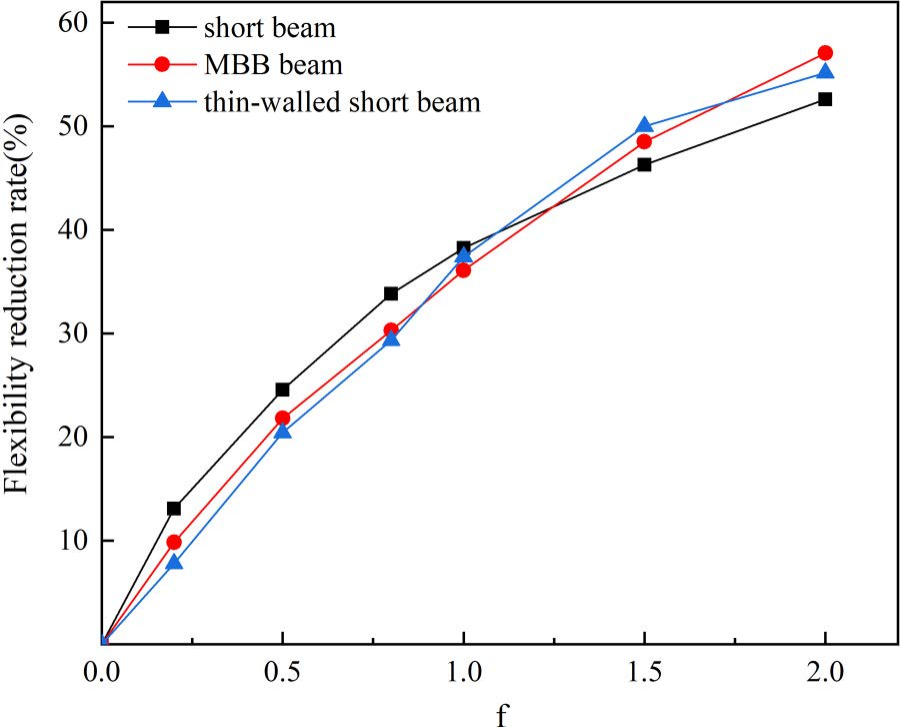
Flexibility reduction rate under different examples.

The flexibility reduction rates under different examples are shown in Fig 12. It can be seen that as *f* increases the structural flexibility continuously decreases, and the increase rate of flexibility reduction rate gradually slows down. The results indicate that the amount of reinforcement material should be increased at appropriate positions to reduce the structural flexibility, and the more reinforcement material used, the more significant the effect.

The optimization results demonstrate the effectiveness of the proposed MMC-SIMP sequential topology optimization method, which can achieve multi-material structural design based on the optimization results. Since the overall effective structure has been determined by the MMC method, the local reinforcement structures obtained by the SIMP method exhibit greater adjustability, facilitating post-processing of discontinuous, relatively isolated, and difficult-to-process structures without fundamentally affecting the overall structural performance.

(2) **Calculation efficiency and accuracy**

The factors that affect computational efficiency mainly include two aspects: one is the design variable scale, which mainly involves sensitivity analysis and variable updates, and the other is the finite element analysis, which mainly involves calculating the stiffness matrix and solving the equilibrium equation. The proposed method adopts sequential topology optimization, using MMC method for structural topology optimization, and then using SIMP method for material layout optimization. The connection between the two optimization methods is achieved by using a surrogate material model to identify mapping elements, without increasing additional computation cost. Therefore, the computational efficiency of the proposed method is determined by the sum of the computational times of the two optimization methods. Since this paper only focuses on the study of two-phase materials, the MMC method has already identified one material, while the SIMP method only needs to determine whether the mapping elements select the second material (i.e., there is only one variable in the SIMP method). Therefore, the SIMP method can use the optimization criteria (OC) method to update the design variables. In terms of calculation accuracy, the accuracy of the proposed method depends on the inherent accuracies of the MMC and SIMP methods.

(3) **Applicability**

Topology optimization of multi-material structures not only requires determining the structural topology, but also the types of materials within the structure. The optimization ideas can be divided into concurrent optimization and sequential optimization. In concurrent optimization [37–41], the optimization of structural topology and material selection is carried out simultaneously. During the optimization, structural topology information and material property information are exchanged in real-time, which affects sensitivity calculation and variable update. In sequential optimization, the structural topology is generally obtained first, and then the distribution of materials within the known structural topology is optimized. During the optimization, structural topology optimization and material selection optimization are performed sequentially. Comparatively speaking, concurrent optimization can naturally achieve superior multi-material structure, but its optimization process is complex and the computational scale of single iteration is large. The selection of materials for sequential optimization is based on the previous structural topology, so the structural topology optimization is not affected by the selection of multi-material.

**Fig 13:**
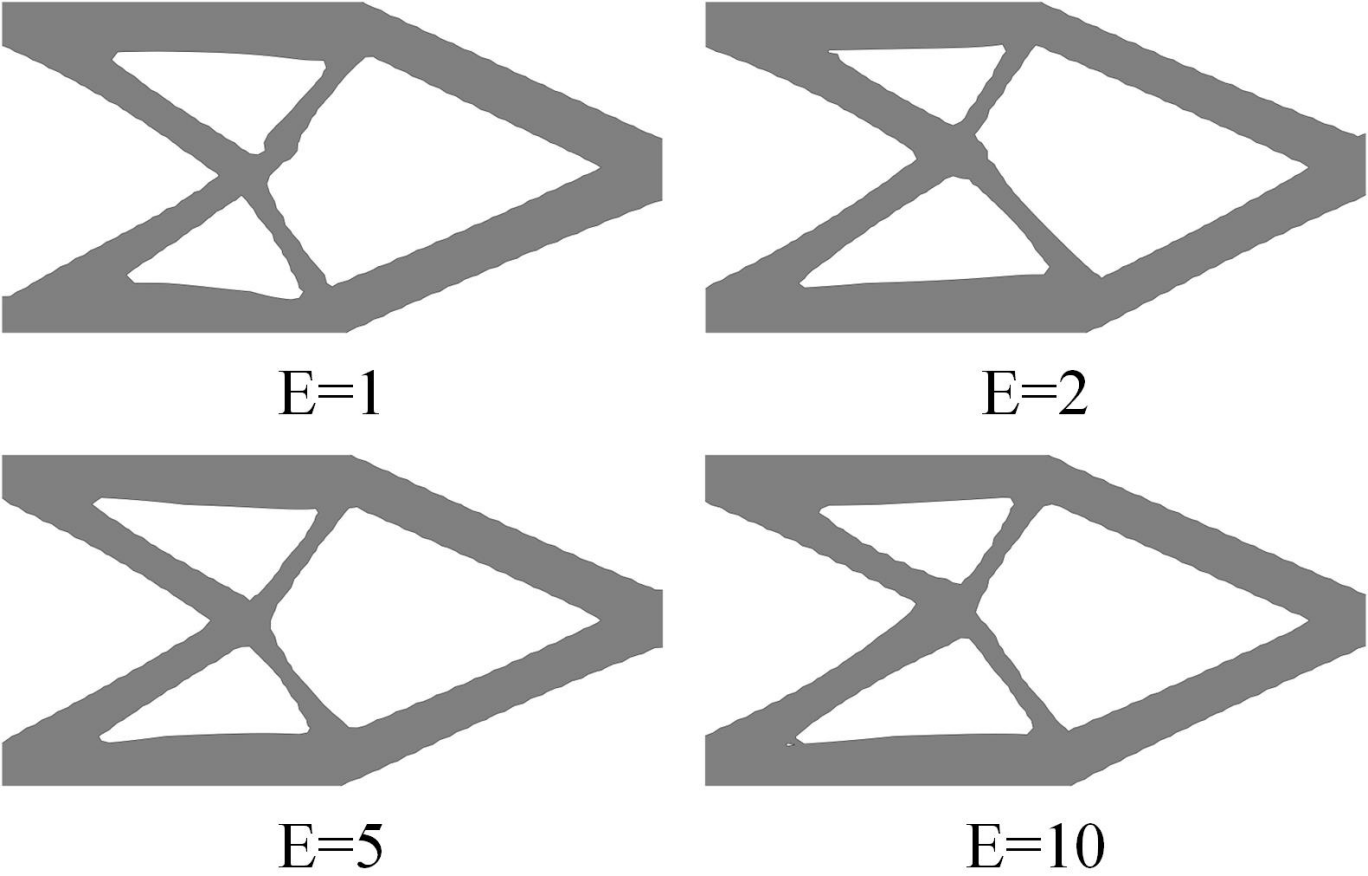
Optimization results of different Young's modulus based on MMC method.

The structural topology obtained by sequential optimization is usually determined by a single material, and the final structure may not be the optimal structure under multiple materials. However, the idea of sequential optimization is easy to implement and the convergence of optimization results is controllable, which also has great application prospects. For example, if the optimized structure of a certain component has been obtained, high-strength and lightweight materials can be partially replaced to further improve structural stiffness and achieve structural lightweighting. Additionally, Fig 13 shows the structural topology obtained by MMC method with different Young’s moduli. It can be seen that the structural topologies obtained by setting different Young’s moduli are quite similar, indicating the influence of different materials on structural topology changes is limited, which also indicates the feasibility of the sequential optimization idea.

## 7. Conclusion

In this paper, an MMC-SIMP sequential topology optimization method for multi-material structural design is proposed based on the combination of the MMC method and the SIMP method. Due to its explicit boundary expression, it can also be regarded as an explicit topology optimization method. In the proposed method, the advantages of both topology optimization methods are fully utilized: the structural topology is determined by the MMC method, while the internal material layout of the structure is determined by the SIMP method. The proposed method not only explicitly expresses the optimized structure but also fully captures the design details, significantly reducing the number of design variables compared to using the SIMP method alone. Meanwhile, the proposed method involves less data exchange between different optimization methods in real-time, making it simple, feasible, and widely applicable. Through numerical examples of two-phase materials at various material volume ratios, it is shown that the proposed method can obtain effective multi-material structures, with clear structural boundaries and internal material layouts, which proves the feasibility of the proposed method.

It should be noted that the structural topology is determined by the MMC method based on a single base material, and other material properties cannot affect the structural topology. Therefore, the optimization degrees of freedom for multi-material structures are limited. However, due to its simplicity and feasibility, this method also has wide application prospects, especially in providing reference for the design of local reinforcement structures for components and further lightweighting of components, such as adding surface coatings or local fiber reinforcement.

## Supporting information

S1 File Matlab codes of an MMC-SIMP sequential topology optimization method based on two-phase materials structure. (DOCX)
