# Supplementary material for: Design of the multi-material structure using an MMC-SIMP sequential topology optimization method
Source: PLoS One. 2025 May 9;20(5):e0321100. doi: 10.1371/journal.pone.0321100 (PMC12063823; doi:10.1371/journal.pone.0321100)
Supplement: S1 File — (DOCX) [file pone.0321100.s001.docx]

function MSS01(DW,DH,nelx,nely,x_int,y_int,ini_val,volfrac,f)

% FEM data initialization

DW=2;DH=1;nelx=80;nely=40;x_int=0.5;y_int=0.5;ini_val=[0.38 0.04 0.06 0.04 0.7];volfrac=0.4;

M = [nely + 1,nelx + 1];

EW = DW / nelx; % length of element

EH = DH / nely; % width of element

[ x ,y ] = meshgrid( EW * (0 : nelx) , EH * ( 0 : nely));

LSgrid.x = x(:);

LSgrid.y = y(:); % coordinate of nodes

% Material properties

h=1; %thickness

E=1;

nu=0.3;

% Component geometry initialization

x0=x_int/2:x_int:DW; % x-coordinates of the centers of components

y0=y_int/2:y_int:DH; % y-coordinates of the centers of components

xn=length(x0); % number of component groups in x direction

yn=length(y0); % number of component groups in y direction

x0=kron(x0,ones(1,2*yn));

y0=repmat(kron(y0,ones(1,2)),1,xn);

N=length(x0); % total number of components in the design domain

L=repmat(ini_val(1),1,N); % vector of the half length of each component

t1=repmat(ini_val(2),1,N); % vector of the half width of component at point A

t2=repmat(ini_val(3),1,N); % vector of the half width of component at point B

t3=repmat(ini_val(4),1,N); % vector of the half width of component at point C

st=repmat([ini_val(5) -ini_val(5)],1,N/2); % vector of the sine value of the inclined angle of each component

variable=[x0;y0;L;t1;t2;t3;st];

%Parameter of MMA

xy00=variable(:);

xval=xy00;

xold1 = xy00;

xold2 = xy00;

%Limits of variable:[x0 ; y0 ; L ; t1 ; t2; t3; st];

xmin=[0 ; 0 ; 0.01 ; 0.01 ; 0.01 ; 0.03; -1.0];

xmin=repmat(xmin,N,1);

xmax=[DW ; DH ; 2.0 ; 0.2 ; 0.2 ; 0.2 ; 1.0];

xmax=repmat(xmax,N,1);

low = xmin;

upp = xmax;

m = 1; %number of constraint

Var_num=7; % number of design variables for each component

nn=Var_num*N;

c=1000*ones(m,1);

d=zeros(m,1);

a0=1;

a=zeros(m,1);

%Define loads and supports(Short beam)

fixeddofs=1:2*(nely+1);

alldofs=1:2*(nely+1)*(nelx+1);

freedofs=setdiff(alldofs,fixeddofs);

loaddof=2*(nely+1)*nelx+nely+2;

F=sparse(loaddof,1,-1,2*(nely+1)* (nelx+1),1);

%Preparation FE analysis

nodenrs=reshape(1:(1+nelx)*(1+nely),1+nely,1+nelx);

edofVec=reshape(2*nodenrs(1:end-1,1:end-1)-1,nelx*nely,1);

edofMat=repmat(edofVec,1,8)+repmat([0 1 2*nely+[2 3 4 5] 2 3],nelx*nely,1);

iK=kron(edofMat,ones(8,1))';

jK=kron(edofMat,ones(1,8))';

EleNodesID=edofMat(:,2:2:8)./2;

iEner=EleNodesID';

[KE] = BasicKe(E,nu, EW, EH,h); % stiffness matrix k^s is formed

%Initialize iteration

p=6;

alpha=1e-3; % parameter alpha in the Heaviside function

epsilon=4*min(EW,EH); % regularization parameter epsilon in the Heaviside function

Phi=cell(N,1);

Loop=1;

change=1;

maxiter=200; % the maximum number of iterations

while change>0.001 && Loop<maxiter

%Forming Phi^s

for i=1:N

Phi{i}=tPhi(xy00(Var_num*i-Var_num+1:Var_num*i),LSgrid.x,LSgrid.y,p);

end

%Union of components

tempPhi_max=Phi{1};

for i=2:N

tempPhi_max=max(tempPhi_max,Phi{i});

end

Phi_max=reshape(tempPhi_max,nely+1,nelx+1);

%Plot components

contour(reshape(x , M), reshape(y , M),Phi_max,[0,0]);

axis equal;axis([0 DW 0 DH]);pause(1e-6);

% Calculating the finite difference quotient of H

H=Heaviside(Phi_max,alpha,nelx,nely,epsilon);

diffH=cell(N,1);

for j=1:N

for ii=1:Var_num

xy001=xy00;

xy001(ii+(j-1)*Var_num)=xy00(ii+(j-1)*Var_num)+max(2*min(EW,EH),0.005);

tmpPhiD1=tPhi(xy001(Var_num*j-Var_num+1:Var_num*j),LSgrid.x,LSgrid.y,p);

tempPhi_max1=tmpPhiD1;

for ik=1:j-1

tempPhi_max1=max(tempPhi_max1,Phi{ik});

end

for ik=j+1:N

tempPhi_max1=max(tempPhi_max1,Phi{ik});

end

xy002=xy00;

xy002(ii+(j-1)*Var_num)=xy00(ii+(j-1)*Var_num)-max(2*min(EW,EH),0.005);

tmpPhiD2=tPhi(xy002(Var_num*j-Var_num+1:Var_num*j),LSgrid.x,LSgrid.y,p);

tempPhi_max2=tmpPhiD2;

for ik=1:j-1

tempPhi_max2=max(tempPhi_max2,Phi{ik});

end

for ik=j+1:N

tempPhi_max2=max(tempPhi_max2,Phi{ik});

end

HD1=Heaviside(tempPhi_max1,alpha,nelx,nely,epsilon);

HD2=Heaviside(tempPhi_max2,alpha,nelx,nely,epsilon);

diffH{j}(:,ii)=(HD1-HD2)/(2*(max(2*min(EW,EH),0.005)));

end

end

%FEA

denk = sum( H(EleNodesID).^2, 2 ) / 4;

den=sum( H(EleNodesID), 2 ) / 4;

A1=sum(den)*EW*EH;

U=zeros(2*(nely+1)*(nely+1),1);

sK = KE(:)*denk(:)';

K = sparse(iK(:),jK(:),sK(:)); K = (K+K')/2;

U(freedofs,:) = K(freedofs,freedofs)\F(freedofs,:);

%Energy of element

energy = sum((U(edofMat)*KE).*U(edofMat),2);

sEner=ones(4,1)*energy'/4;

energy_nod=sparse(iEner(:),1,sEner(:));

Comp=F'*U;

% Sensitivities

df0dx=zeros(Var_num*N,1);

dfdx=zeros(Var_num*N,1);

for k=1:N

df0dx(Var_num*k-Var_num+1:Var_num*k,1)=2*energy_nod'.*H*diffH{k};

dfdx(Var_num*k-Var_num+1:Var_num*k,1)=sum(diffH{k})/4;

end

%MMA optimization

f0val =Comp;

df0dx=-df0dx/max(abs(df0dx));

fval=A1/(DW*DH)-volfrac;

dfdx=dfdx/max(abs(dfdx));

% [xmma,ymma,zmma,lam,xsi,eta,mu,zet,ss,low,upp] = ...

% mmasub1(m,nn,Loop,xval,xmin,xmax,xold1,xold2, ...

% f0val,df0dx,fval,dfdx,low,upp,a0,a,c,d);

df0dx2=0;

dfdx2=0;

[xmma,low,upp] =mmasub1(m,nn,Loop,xval,xmin,xmax,xold1,xold2,df0dx,df0dx2,fval,dfdx,dfdx2,low,upp,a0,a,c,d);

xold2 = xold1;

xold1 = xval;

change=max(abs(xval-xmma));

xval = xmma;

xy00=round(xval*1e4)/1e4;

disp([' It.: ' sprintf('%4i\t',Loop) ' Obj.: ' sprintf('%6.3f\t',f0val) ' Vol.: ' ...

sprintf('%6.4f\t',fval) 'ch.:' sprintf('%6.4f\t',change)]);

Loop = Loop + 1;

end

f=1.5;

change01=1;

loop01=0;

penal=3;

E1=E; E2=2;

[NKE] = NBasicKe(nu, EW, EH,h); % stiffness matrix k^s is formed

Ei=find(denk>alpha^2);

nn=length(Ei);

Svariable(1:nn,1)=volfrac;

Sxy00=Svariable(:);

while change01>0.01

loop01=loop01+1;

xoldS=Sxy00;

SE=Sxy00.^penal*E1+(1-Sxy00.^penal)*E2;

Ndenk=denk(:).^E1;

for ia=1:nn

Ndenk(Ei(ia))=denk(Ei(ia))*SE(ia);

end

NsK = NKE(:)*Ndenk(:)';

NK = sparse(iK(:),jK(:),NsK(:)); NK = (NK+NK')/2;

NU=zeros(2*(nely+1)*(nely+1),1);

NU(freedofs,:) = NK(freedofs,freedofs)\F(freedofs,:);

Comp01=F'*NU;

dc=zeros(nn,1);

for ib=1:nn

UU=NU(edofMat(Ei(ib),:));

dc(ib)=penal* Sxy00(ib).^(penal-1)*(E1-E2)* UU'*NKE* UU;

%dc(ib)=denk(Ei(ib))*penal* Sxy00(ib).^(penal-1)*(E1-E2)* UU'*NKE* UU;

end

col(1:nely,1:nelx)=0;

col=col(:);

for ic=1:nn

col(Ei(ic))=Sxy00(ic);

end

col=reshape(col,nely,nelx);

rmin=2;

dc01=zeros(nely*nelx,1);

for ie=1:nn

dc01(Ei(ie))=dc(ie);

end

dc01=reshape(dc01,nely,nelx);

dc01 = Sfilter(nelx,nely,rmin,col,dc01);

dc02=dc01(:);

dc03=zeros(nn,1);

for ig=1:nn

dc03(ig)=dc02(Ei(ig));

end

Sxy00=OC(nn,Sxy00,f,dc03);

change01 = max(max(abs(Sxy00-xoldS)));

disp([' It.: ' sprintf('%4i',loop01) ' Obj.: ' sprintf('%10.4f',Comp01) ...

' Vol.: ' sprintf('%6.3f',sum(sum(Sxy00))/sum(den)) ...

' ch.: ' sprintf('%6.3f',change01 )])

% PLOT DENSITIES

colormap(gray); imagesc(-col); axis tight; axis off; pause(1e-6);

end

ends

%Forming Phi_i for each component

function [tmpPhi]=tPhi(xy,LSgridx,LSgridy,p)

st=xy(7);

ct=sqrt(abs(1-st*st));

x1=ct*(LSgridx - xy(1))+st*(LSgridy - xy(2));

y1=-st*(LSgridx - xy(1))+ct*(LSgridy - xy(2));

bb=(xy(5)+xy(4)-2*xy(6))/2/xy(3)^2*x1.^2+(xy(5)-xy(4))/2*x1/xy(3)+xy(6);

tmpPhi= -((x1).^p/xy(3)^p+(y1).^p./bb.^p-1);

end

%Heaviside function

function H=Heaviside(phi,alpha,nelx,nely,epsilon)

num_all=(1:(nelx+1)*(nely+1))';

num1=find(phi>epsilon);

H(num1)=1;

num2=find(phi<-epsilon);

H(num2)=alpha;

num3=setdiff(num_all,[num1;num2]);

H(num3)=3*(1-alpha)/4*(phi(num3)/epsilon-phi(num3).^3/(3*(epsilon)^3))+(1+alpha)/2;

end

%Element stiffness matrix

function [NKE] = NBasicKe(nu, a, b,h)

k = [-1/6/a/b*(nu*a^2-2*b^2-a^2), 1/8*nu+1/8, -1/12/a/b*(nu*a^2+4*b^2-a^2), 3/8*nu-1/8, ...

1/12/a/b*(nu*a^2-2*b^2-a^2),-1/8*nu-1/8, 1/6/a/b*(nu*a^2+b^2-a^2), -3/8*nu+1/8];

NKE =h/(1-nu^2)*...

[ k(1) k(2) k(3) k(4) k(5) k(6) k(7) k(8)

k(2) k(1) k(8) k(7) k(6) k(5) k(4) k(3)

k(3) k(8) k(1) k(6) k(7) k(4) k(5) k(2)

k(4) k(7) k(6) k(1) k(8) k(3) k(2) k(5)

k(5) k(6) k(7) k(8) k(1) k(2) k(3) k(4)

k(6) k(5) k(4) k(3) k(2) k(1) k(8) k(7)

k(7) k(4) k(5) k(2) k(3) k(8) k(1) k(6)

k(8) k(3) k(2) k(5) k(4) k(7) k(6) k(1)];

end

% MMA

function [xmma,low,upp] = ...

mmasub1(m,n,iter,xval,xmin,xmax,xold1,xold2, ...

df0dx,df0dx2,fval,dfdx,dfdx2,low,upp,a0,a,c,d)

epsimin = 10^(-3);

feps = 0.000001;

asyinit = 0.1;

asyincr = 1.2;

asydecr = 0.7;

albefa = 0.4;

een = ones(n,1);

zeron = zeros(n,1);

% %Calculation of the asymptotes low and upp %%

if iter <= 2

low = xval - asyinit*(xmax-xmin);

upp = xval + asyinit*(xmax-xmin);

end

if iter >= 3

xxx = (xval-xold1).*(xold1-xold2);

factor = een;

factor(xxx > 0) = asyincr;

factor(xxx < 0) = asydecr;

low = xval - factor.*(xold1 - low);

upp = xval + factor.*(upp - xold1);

end

%% Calculation of the bounds alfa and beta %%

xxx = low + albefa*(xval-low);

alfa = max(xxx,xmin);

xxx = upp - albefa*(upp-xval);

beta = min(xxx,xmax);

%% Calculations of p0, q0, P, Q and b %%

ux1 = upp-xval;

ux2 = ux1.*ux1;

ux3 = ux2.*ux1;

xl1 = xval-low;

xl2 = xl1.*xl1;

xl3 = xl2.*xl1;

ul1 = upp-low;

ulinv1 = een./ul1;

uxinv1 = een./ux1;

xlinv1 = een./xl1;

uxinv3 = een./ux3;

xlinv3 = een./xl3;

diap = (ux3.*xl1)./(2*ul1);

diaq = (ux1.*xl3)./(2*ul1);

p0 = zeron;

p0(df0dx > 0) = df0dx(df0dx > 0);

p0 = p0 + 0.001*abs(df0dx) + feps*ulinv1;

p0 = p0.*ux2;

q0 = zeron;

q0(df0dx < 0) = -df0dx(df0dx < 0);

q0 = q0 + 0.001*abs(df0dx) + feps*ulinv1;

q0 = q0.*xl2;

dg0dx2 = 2*(p0./ux3 + q0./xl3);

del0 = df0dx2 - dg0dx2;

delpos0 = zeron;

delpos0(del0 > 0) = del0(del0 > 0);

p0 = p0 + delpos0.*diap;

q0 = q0 + delpos0.*diaq;

P = zeros(m,n);

P(dfdx > 0) = dfdx(dfdx > 0);

P = P * diag(ux2);

Q = zeros(m,n);

Q(dfdx < 0) = -dfdx(dfdx < 0);

Q = Q * diag(xl2);

dgdx2 = 2*(P*diag(uxinv3) + Q*diag(xlinv3));

del = dfdx2 - dgdx2;

delpos = zeros(m,n);

delpos(del > 0) = del(del > 0);

P = P + delpos*diag(diap);

Q = Q + delpos*diag(diaq);

b = P*uxinv1 + Q*xlinv1 - fval ;

end

% OC

function [xnew]=OC(nn,x,f,dc)

l1 = 0; l2 = 100000; move = 0.2;

while (l2-l1 > 1e-4)

lmid = 0.5*(l2+l1);

xnew = max(0.001,max(x-move,min(1.,min(x+move,x.*sqrt(-dc./lmid)))) );

%xnew(find(passive)) = 0.001;

if sum(sum(xnew)) - nn/(1+f) > 0;

l1 = lmid;

else

l2 = lmid;

end

end

% Sensitivity filtering

function [dcn]=Sfilter(nelx,nely,rmin,x,dc)

dcn=zeros(nely,nelx);

for i = 1:nelx

for j = 1:nely

sum=0.0;

for k = max(i-floor(rmin),1):min(i+floor(rmin),nelx)

for l = max(j-floor(rmin),1):min(j+floor(rmin),nely)

fac = rmin-sqrt((i-k)^2+(j-l)^2);

sum = sum+max(0,fac);

dcn(j,i) = dcn(j,i) + max(0,fac)*x(l,k)*dc(l,k);

end

end

dcn(j,i) = dcn(j,i)/(x(j,i)*sum);

end

end
